# Fast imputation using medium or low-coverage sequence data

**DOI:** 10.1186/s12863-015-0243-7

**Published:** 2015-07-14

**Authors:** Paul M. VanRaden, Chuanyu Sun, Jeffrey R. O’Connell

**Affiliations:** Animal Genomics and Improvement Laboratory, Agricultural Research Service, United States Department of Agriculture, Beltsville, MD 20705-2350 USA; National Association of Animal Breeders, Columbia, Missouri 65205 USA; University of Maryland School of Medicine, Baltimore, Maryland 21201 USA

**Keywords:** Imputation, Genotype, Sequence read depth, Allele probability

## Abstract

**Background:**

Accurate genotype imputation can greatly reduce costs and increase benefits by combining whole-genome sequence data of varying read depth and array genotypes of varying densities. For large populations, an efficient strategy chooses the two haplotypes most likely to form each genotype and updates posterior allele probabilities from prior probabilities within those two haplotypes as each individual’s sequence is processed. Directly using allele read counts can improve imputation accuracy and reduce computation compared with calling or computing genotype probabilities first and then imputing.

**Results:**

A new algorithm was implemented in findhap (version 4) software and tested using simulated bovine and actual human sequence data with different combinations of reference population size, sequence read depth and error rate. Read depths of ≥8× may be desired for direct investigation of sequenced individuals, but for a given total cost, sequencing more individuals at read depths of 2× to 4× gave more accurate imputation from array genotypes. Imputation accuracy improved further if reference individuals had both low-coverage sequence and high-density (HD) microarray data, and remained high even with a read error rate of 16 %. With read depths of ≤4×, findhap (version 4) had higher accuracy than Beagle (version 4); computing time was up to 400 times faster with findhap than with Beagle. For 10,000 sequenced individuals plus 250 with HD array genotypes to test imputation, findhap used 7 hours, 10 processors and 50 GB of memory for 1 million loci on one chromosome. Computing times increased in proportion to population size but less than proportional to number of variants.

**Conclusions:**

Simultaneous genotype calling from low-coverage sequence data and imputation from array genotypes of various densities is done very efficiently within findhap by updating allele probabilities within the two haplotypes for each individual. Accuracy of genotype calling and imputation were high with both simulated bovine and actual human genomes reduced to low-coverage sequence and HD microarray data. More efficient imputation allows geneticists to locate and test effects of more DNA variants from more individuals and to include those in future prediction and selection.

## Background

Genotype imputation greatly reduces cost and increases accuracy of estimating genetic effects by increasing the ratio of output to input data, but algorithms must be efficient because numbers of genotypes to impute may increase faster than computer resources. International exchange of sequence data has provided whole genomes for thousands of humans [1] and hundreds of bulls [2]. Array genotypes are also available for hundreds of thousands of both humans and cattle, and all of these could be imputed to sequence with varying accuracy and difficulty depending on the algorithms used. Especially for rare alleles, accurate estimation of effects requires large numbers of individuals with phenotypes and observed or imputed genotypes.

Genome sequencing directly interrogates the genetic variation that underlies quantitative traits and disease susceptibility and thus enables a better understanding of biology. However, deeply sequencing large numbers of individuals is still not practical given current costs [3]. Instead, sequence genotypes can be imputed accurately by combining information from individuals sequenced at lower coverage and from those genotyped with less expensive single nucleotide polymorphism (SNP) arrays. Sequencing and genotyping projects always involve a tradeoff between number of individuals sequenced and read depth or SNP density. More efficient imputation will allow geneticists to locate and test effects of more DNA variants from more individuals and to include those in future prediction and selection.

In many livestock and some crop populations, most DNA may be inherited from a small group of common ancestors a few generations in the past. If most ancestors are sequenced and phased, their large segments of chromosome can be traced to descendants using HD SNP arrays [4, 5]. Beagle (version 3) [6] and IMPUTE2 [7] provide good results in cattle without using pedigree data, except for single nucleotide variants (SNVs) with low frequency [8, 9]. Beagle (version 4) can input genotype probabilities instead of only known genotypes and is tested here for processing low-coverage sequence data.

Animal breeders have developed imputation software that runs many times faster than software from human geneticists by using general pedigrees, long-range phasing and the high degree of haplotypes shared across very large populations [10–12]. For example, programs such as findhap and FImpute use genotypes from several low-density arrays to impute >60,000 markers for >500,000 dairy cattle in monthly or weekly national genomic evaluation systems [13] and can impute >600,000 markers for >100,000 animals in 10 h using 6 processors [14]. These rapid methods also can give higher imputation accuracy to sequence data [15]. Similarly, plant breeders have developed fast and accurate imputation algorithms for low-coverage sequence data from inbred populations [16].

Human geneticists have not used general pedigrees in imputation until very recently [17] and could benefit from efficient algorithms already available for large, related populations. Use of pedigree can improve speed and imputation accuracy, especially for recent mutations, because the normal and mutated versions of a haplotype are both present in the same population. Tracing inheritance within families separates the normal from the mutated haplotype. Animal breeding datasets often have 10 consecutive generations of genotyped ancestors to track the occurrence and inheritance of such mutations [18, 19]. Knowing the four haplotypes of the two parents or the haplotypes of grandparents reduces the search space from thousands to just a few haplotypes plus potential crossovers.

Sequencing more individuals at lower coverage often provides more power for equivalent cost, which is proportional to number of individuals times coverage depth [20–22]. However, imputation algorithms for sequence data with low read depth or high error must account for less certain knowledge of homozygotes and heterozygotes than in array genotypes [23–26]. At each particular locus in diploids, either of the two alleles may be read a different number of times, not read at all or misread, which requires additional probability calculations not needed in many previous algorithms that assumed genotypes were known [27]. With low read depth, matching to HD genotypes is less accurate, but imputation can be improved by including HD genotypes also for the sequenced individuals so that known genotypes are available at corresponding loci [24].

Without imputation, achieving a sensitivity of 99 % for detecting heterozygotes would require an average sequencing depth of >21× after accounting for sequencing errors [28]. Current sequence reads are mostly 100 to 200 bases in length and contain only one or two variants per segment. The natural phasing that could occur when reading linked alleles [27] does not occur often because the segments are too short to contain multiple heterozygous loci. Genotypes can be imputed accurately from sequence data with low read depth or high error, but new strategies are needed to efficiently impute many thousands of whole genomes [29]. A simple strategy without imputation can identify variants of interest, but their estimated effect sizes are biased downward [30].

This study (1) adapts the fast imputation algorithms of VanRaden et al. [14] to process sequence read depths directly instead of first calling genotypes, (2) tests the accuracy of genotype calling and imputation for different reference population sizes, sequence read depths and error rates and (3) compares including or excluding SNP array data of different densities in the data for the sequenced individuals. The tests used simulated bovine and actual human sequences.

## Results

Computing costs are low for version 4 of findhap [31] and increase in linear proportion to population size but less than proportionally to SNV density (Table 1). Computing times for findhap ranged from 11 min with 500 individuals (250 sequences plus 250 HD genotypes) to 7 h with 10,000 sequences. With 500 individuals, Beagle took almost 3 days (387 times longer than findhap) and was not tested for larger populations. The timing tests for both findhap and Beagle used 10 processors and one simulated bovine chromosome. With SNV densities of 2000, 20,000 and 1 million evenly spaced SNVs across the chromosome, computing times for findhap increased with ratios of only 1 to 4.25 to 105, respectively, compared with SNV density ratios of 1 to 10 to 500. Efficiency of findhap improves with higher SNV density because haplotype windows contain more SNVs, but most haplotypes can be excluded after checking only a few SNVs.

**Table 1:** Computing times with 10 processors for different populations and SNV densities using one simulated bovine chromosome

Software	Reference data		Array genotypes		Computing time (minutes)
	Population size	SNVs	Population size	SNVs	
Beagle	250	1,000,000	250	20,000	4258
findhap	250	1,000,000	250	20,000	11
	500	1,000,000	250	20,000	19
	1000	1,000,000	250	20,000	37
	10,000	1,000,000	250	20,000	420
	10,000	20,000	250	2000	17
	10,000	2000	250	200	4
	884	394,274	218	39,440	17

Compared with version 2 of findhap [11], version 4 requires only slightly more time but almost twice as much memory because of storing 2-byte probabilities instead of 1-byte haplotype codes. Processing one chromosome required about 5 Gigabytes of memory for 1000 sequenced animals plus 250 animals with HD genotypes. The memory required depends on the maximum number of haplotypes in any segment and may also scale less than linearly with population size. Version 3 of findhap [31] has nearly the same performance as version 2 for initial processing of data, but can use prior haplotypes to speed processing when new genotypes are added [32]. It inputs the previous list of haplotypes sorted by descending frequency and the numeric codes that specify which two haplotypes make up the genotype of each animal. Those features were retained in version 4 and improve speed, especially when the new genotypes are from progeny of the previous population.

Beagle and findhap imputation accuracies are compared in Table 2. When read depth for sequenced animals was 8×, both imputation methods were highly accurate for genotype calling, and Beagle had 1 or 2 % more correct calls when imputing from HD. However, when read depth was ≤4×, findhap became much more accurate than Beagle for both genotype calling from sequence and imputation from HD. Beagle software first performs genotype calling for the sequenced population and then imputation for the HD population, whereas findhap processes the two populations together. The combined data allows the HD population to improve the accuracy of phasing the sequenced population in findhap, but the HD population makes no contribution with Beagle. Accuracy of Beagle might be improved using more than the default number of iterations [4] but at the expense of slower computation. Accuracy of findhap might be improved by including more family members with HD, but that was not tested here.

**Table 2:** Accuracy and percentage correct for calling genotypes of sequenced animals and for imputation for 250 young animals with simulated bovine data

Sequenced population		Software	Genotype calling (1 % error rate)		Imputation from HD to sequence	
Size	Coverage		Percentage correct	Correlation	Percentage correct	Correlation
250	8×	Findhap	98.7	0.981	95.0	0.926
		Beagle	99.0	0.984	97.1	0.956
500	8×	Findhap	99.1	0.988	98.1	0.974
		Beagle	99.6	0.994	99.4	0.991
250	4×	Findhap	95.8	0.939	93.1	0.897
		Beagle	95.0	0.918	78.2	0.582
250	2×	Findhap	91.3	0.879	89.2	0.837
		Beagle	79.5	0.602	63.5	0.100

**Fig. 1:**
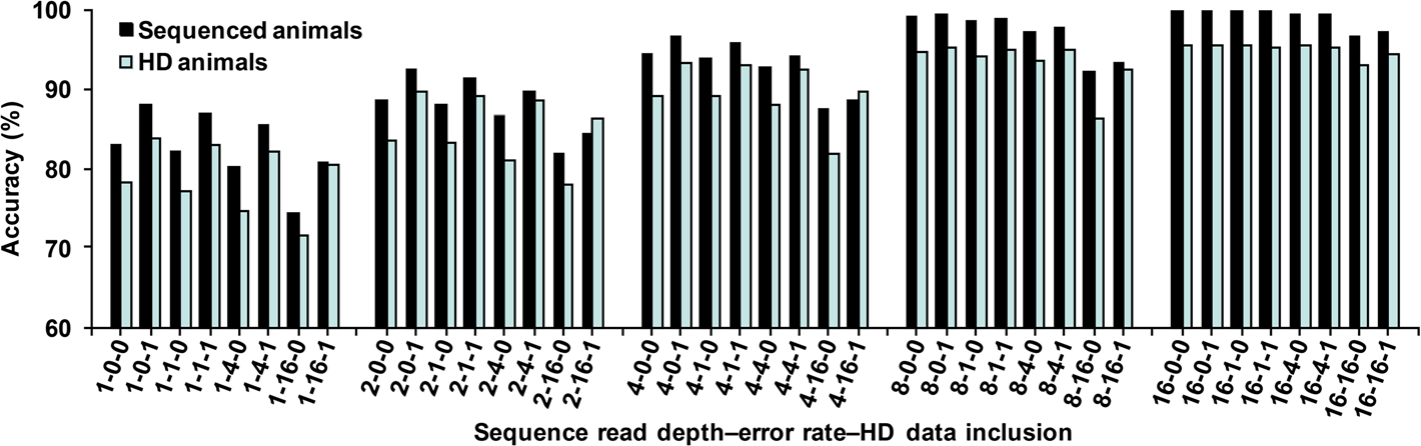
Correctly called or imputed genotypes by read depth, error rate, and HD inclusion. The percentage of correctly called genotypes is shown for 250 sequenced animals as well as the percentage of correctly imputed SNPs for 250 animals with HD genotypes. Genotype calling and imputation were done simultaneously. The x-axis shows sequence read depth, error rate and inclusion (1) or exclusion (0) of HD data for sequenced animals (e.g. 2-4-1 indicates a sequence read depth of 2×, error rate of 4 %, and the inclusion of HD data in the simulated sequence data)

Accuracies from findhap for both genotype calling and imputation improved if the sequenced animals also had array genotypes (Fig. 1). With 250 sequenced and 250 HD genotyped animals and a sequencing error rate of 1 %, the accuracy for sequenced animals was 82.3, 87.9, 93.9, 98.5 and 99.9 % for read depths of 1×, 2×, 4×, 8× and 16×, respectively, when their data did not include HD genotypes and 87.1, 91.3, 95.8, 98.7 and 99.9 % when their data did include HD genotypes. For animals with only HD data, corresponding imputation accuracy was 77.2, 83.3, 89.1, 94.3 and 95.4 % when data for sequenced animals did not include HD genotypes and 83.1, 89.2, 93.1, 95.0 and 95.3 % when they did. Including HD data improved genotype calling and imputation accuracy, especially when sequence read depth was lower or sequencing error rate was higher. Even with an error rate of 0 % for sequencing, higher coverage or inclusion of HD genotypes improves accuracy because homozygous genotypes cannot be directly determined from low-coverage sequence data.

Larger population size also improved accuracy of genotype calling for sequenced animals (Table 3), as expected. Gains were largest for low read depth and high error rate. With a read depth of 2× and error rate of 1 %, accuracy using findhap with 250, 500 and 1000 sequenced animals was 91.3, 94.1 and 95.9 %, respectively (Tables 2 and 3). With an error rate of ≤1%, a larger population improved genotype calling slightly for 8× but not for 16× coverage, because calling accuracy was already ≥99.9 % with populations of 250, 500 or 1000 animals (Fig. 1 and Table 3). However, accuracy did improve with population size if error rates were higher.

**Table 3:** Percentages of genotypes called correctly by findhap for simulated sequenced animals

Error rate (%)	Population size	Sequence read depth				
		1×	2×	4×	8×	16×
0	500	92.1	94.9	97.7	99.5	100.0
	1000	94.6	96.4	98.3	99.7	100.0
1	500	91.2	94.1	97.0	99.2	99.9
	1000	93.8	95.9	97.8	99.5	99.9
4	500	90.5	93.1	96.1	98.4	99.7
	1000	93.6	95.2	97.2	98.9	99.8
16	500	87.3	89.2	91.6	95.2	98.1
	1000	91.3	92.1	93.6	96.5	98.8

Table 4 shows the accuracy of imputation from HD array to sequence for 250 animals with genotypes from a simulated 600,000 (600 K) SNP array by reference population size, sequence read depth and error rate. Using 500 animals with 16× coverage and 0 % error as the reference population, 98.4 % of imputed genotypes were correct with the new algorithm compared to 97.8 % with version 2 of findhap [14]. With 4× instead of 16× coverage, 96.8 % of imputed genotypes were correct. Using 1000 animals with 16× coverage and 0 % error, 99.3 % of genotypes were imputed correctly from HD compared with 98.4 % using 4 × .

**Table 4:** Accuracy of imputation and percentages of correctly imputed genotypes for 250 animals with genotypes from a simulated 600 K array

Reference population size	Sequence read depth	Error rate (%)							
		0		1		4		16	
		Percentage correct	Correlation	Percentage correct	Correlation	Percentage correct	Correlation	Percentage correct	Correlation
500	16×	98.4	0.978	98.4	0.977	98.3	0.976	97.8	0.969
	8×	98.2	0.975	98.1	0.974	98.0	0.972	96.9	0.956
	4×	96.8	0.956	96.7	0.954	96.8	0.955	95.4	0.934
	2×	94.7	0.925	94.1	0.917	94.2	0.917	93.6	0.909
	1×	90.9	0.869	89.8	0.853	90.0	0.855	90.0	0.855
1000	16×	99.3	0.990	99.2	0.989	99.2	0.989	98.9	0.984
	8×	99.2	0.988	99.1	0.987	99.0	0.986	98.0	0.972
	4×	98.4	0.978	98.2	0.975	98.1	0.973	96.9	0.956
	2×	97.0	0.958	96.5	0.951	96.7	0.953	95.8	0.939
	1×	94.8	0.925	93.8	0.911	93.9	0.912	93.4	0.903
10,000	2×			97.5	0.964				
	1×			95.8	0.939				

Sequence reads with 1 or 4 % error rates reduced imputation success slightly, but 16 % error caused larger reductions (Table 4). For example, with 500 sequenced animals and 8× coverage in the reference population, the HD animals had 98.2, 98.1, 98.0 and 96.9 % correct imputation if sequence error was 0, 1, 4 and 16 %, respectively. With lower read depths, error rates caused larger differences in imputation success, but with 1000 animals and 16× coverage, 98.9 % of genotypes for HD animals were imputed correctly even if the read error rate was 16 %. With 10,000 animals sequenced with 1 % error and 2× or 1× coverage, the imputed genotypes for HD animals were 97.5 and 95.8 % correct, respectively. Accuracies were a little lower if measured by correlation of imputed and true genotypes after centering for allele frequency, but all conclusions were consistent.

**Fig. 2:**
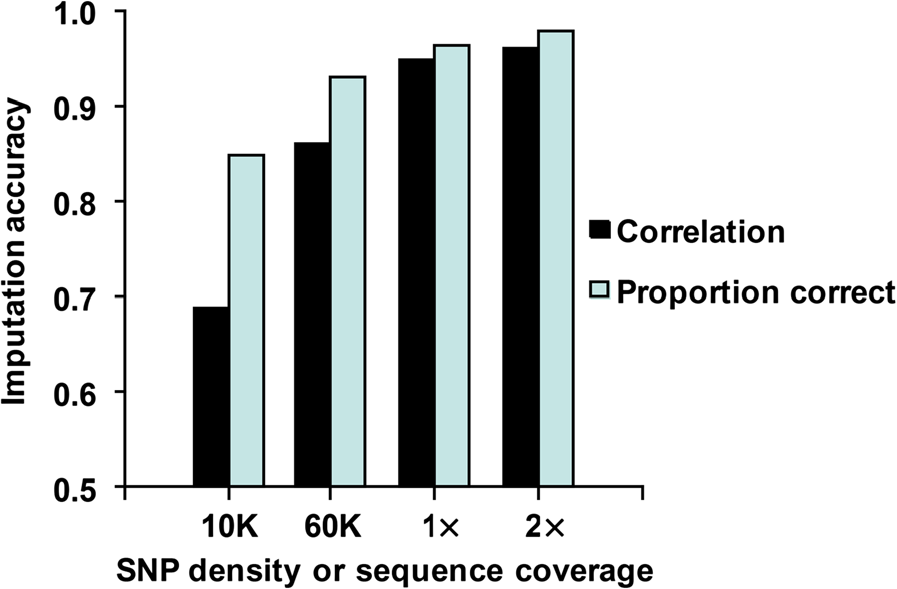
Imputation accuracy for low-density or low-coverage genotypes. Correlations between imputed and true genotypes and proportions of correctly imputed genotypes are shown for 250 animals with low-density SNP or low-coverage sequence genotypes based on a reference population of 500 animals sequenced with a simulated sequence read depth of 8× and 1 % error

Lower density arrays further reduced imputation accuracy. When the reference population had 1000 animals sequenced with 8× coverage, 1 % error and HD markers included, animals genotyped with only a simulated 60,000 (60 K) SNP array had 96.8 % correct imputation and those genotyped with a simulated 10,000 (10 K) SNP array had only 91.7 % correct imputation. In these tests, maximum haplotype length in findhap was extended to 100,000 variants from the 50,000 used for imputing from 600 K SNPs so that sufficient markers were included within each interval. If the animals used to test imputation had low-coverage sequences instead of low-density chips, animals had 99.1 % correct imputation from 2× coverage or 99.0 % correct from 1× coverage with the same reference population (results not shown). Figure 2 shows corresponding imputation accuracies that were somewhat lower when the reference population had 500 animals sequenced at 8× coverage.

When older animals with 8× coverage and younger animals with 2× or 1× coverage or 600 K, 60 K or 10 K SNP genotypes were all included in one data set instead of testing the young animal data types separately, imputation accuracy decreased slightly in some cases because haplotype lengths and other options were not optimal for all data types. Further research is needed to improve these strategies and the resulting accuracy of fast imputation when combining low-density arrays with low-coverage sequence data. Two-stage imputation strategies [5, 14] could improve accuracy for that case.

**Fig. 3:**
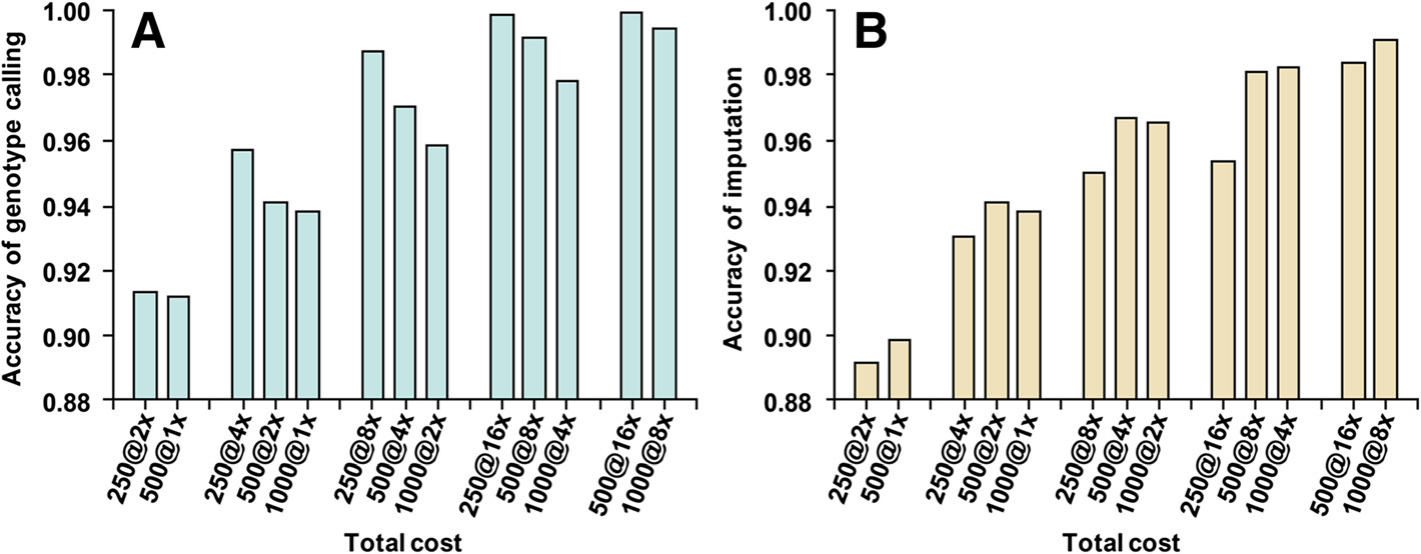
Comparison of correctly called or imputed genotypes based on cost. The proportion of genotypes called correctly from sequence data (**a**) or imputed correctly from HD SNP (**b**) is shown for a given total cost based on simulated reference population size times sequence read depth (e.g. a population size of 250 animals and 2× coverage has the same cost as a population size of 500 animals and 1× coverage)

For the same total cost of genotyping (number of animals times coverage), higher read depth resulted in more accurate genotype calling of sequenced animals, but imputation from HD was more accurate with lower depth and a larger population (Fig. 3). For sequenced animals, accuracy of genotype calling was highest for 250 animals with 16× coverage and declined for 500 with 8× or 1000 with 4× coverage. For imputation from HD, sequencing twice as many animals at half the read depth often resulted in more correct calls (e.g. 95.3 % using 250 animals with 16×, 98.1 % using 500 animals with 8× or 98.2 % using 1000 animals with 4× coverage). However, coverage less than 4× did not further improve imputation. For example, with an error rate of 1 %, accuracy for 1000 animals with 2× coverage was lower than for 500 animals with 4×, and accuracy for 10,000 animals with 2× coverage was lower than for 1000 animals with 8× (Table 4).

**Fig. 4:**
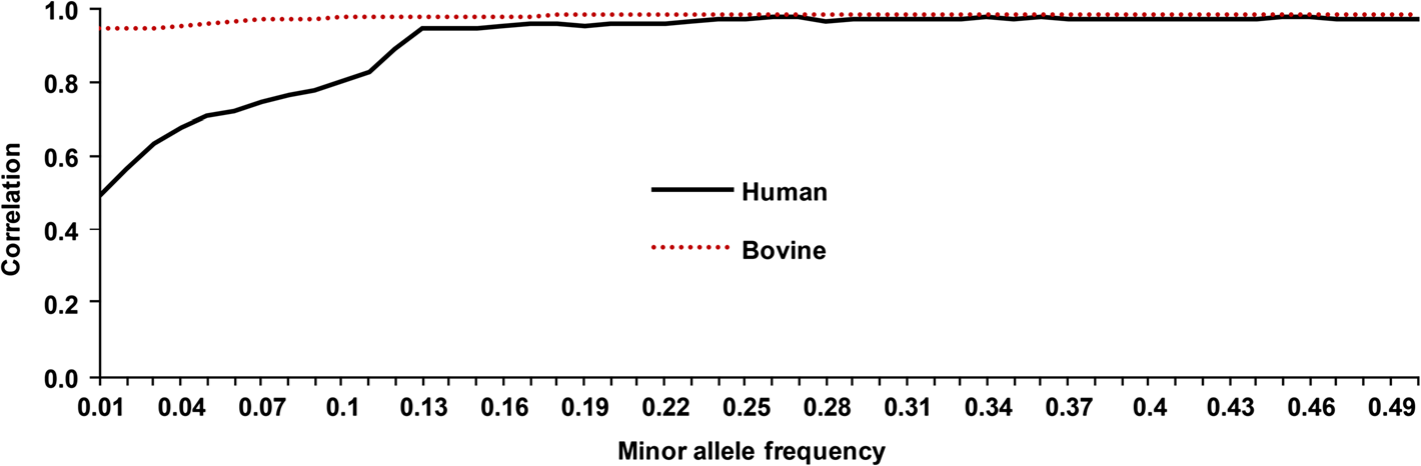
Average correlations of true and imputed sequence variants for human or cattle simulated HD genotypes. Correlations are plotted by minor allele frequency. The 884 humans and 500 cattle with sequence data had 16× coverage with 1 % read errors and included HD data

After reducing coverage and introducing read errors, human chromosome-22 sequences had a pattern for imputation accuracy (Table 5) that was similar to that for simulated bovine sequences. However, the options in findhap were set to a minimum length of 100 SNVs, a maximum length of 20,000 SNVs and processing with 6 iterations per step. In all cases, error rates assumed in the options file equaled the true error rates used in generating the data. After imputing the 90 % missing SNVs from the 10 % observed HD SNVs, >97 % of the sequence genotypes were correct when read depth was ≥8×. For sequenced individuals with ≥8× coverage, >99 % of genotypes were called correctly if error rate was ≤4%. Similar results from both human and simulated bovine data indicate that 500 to 1000 sequences give excellent imputation if coverage is high (e.g. ≥8×). As expected, correlations between imputed and true genotypes (Fig. 4) were lower for variants with low minor allele frequency (MAF) compared with high-MAF variants for both human and cattle data.

**Table 5:** Percentage of genotypes on human chromosome 22 correctly called from various sequence read depths and error rates or imputed correctly from HD

Sequence read depth	Error rate (%)							
	0		1		4		16	
	Sequence	HD	Sequence	HD	Sequence	HD	Sequence	HD
16×	100.0	97.4	99.9	97.4	99.8	97.4	98.9	97.3
8×	99.6	97.3	99.4	97.3	99.0	97.2	98.1	97.2
4×	98.6	96.8	98.3	96.9	97.9	96.9	96.9	96.7
2×	97.0	96.2	96.9	96.3	96.4	96.0	95.1	95.5
1×	95.1	95.2	95.1	95.1	94.5	94.6	93.2	93.8

## Discussion

Software design is becoming more important when processing more data types and rapidly expanding datasets. The new algorithm directly uses read depth counts for the reference and alternate alleles, array genotypes, and general pedigrees to impute sequence genotypes. When read depth was ≤4×, findhap was more accurate than Beagle for both genotype calling and imputation, whereas Beagle was somewhat more accurate when read depth was ≥8×. Computing times were up to 400 times faster with findhap than with Beagle for the pedigreed cattle population.

Advantages might be smaller for human populations without pedigree data, but findhap computing times and imputation accuracy were very similar with or without pedigree for the cattle sequence data, which agrees with results from FImpute that use of pedigree helps mainly at the lowest densities [15]. Beagle has an option to process duos and trios, but this further decreased its speed by about 5 times and increased the memory required. The computing times reported here include both phasing and imputing, and times can be reduced if only imputation is done using a pre-phased reference population in either findhap or Beagle. However, if the quality of genotype calling is greatly reduced in Beagle with low-coverage sequence, then the quality of its phasing likely is also greatly reduced. Wang et al. [25] reported that accuracies and speed of genotype calling with SNPTools were similar to Beagle with 4× data and no pedigree. The much faster speed of FImpute and findhap is mainly because of direct searching for long haplotypes in frequency order instead of using the hidden Markov process of most other programs [33].

Sequencing studies can improve imputation and reduce error rates by obtaining HD genotypes from the same DNA with little added cost [34]. Our results confirmed that the accuracy of genotype calling and imputation increased when read depth was ≤4× and sequenced individuals also had HD genotypes, which was consistent with results of Menelaou and Marchini [24]. Including both HD and low-coverage data for the same individual improved the probability of selecting the two correct haplotypes before updating the allele probabilities within the two selected haplotypes. When animals with only low or medium-density array genotypes were included, accuracy decreased slightly because findhap parameter values were not optimal for all data inputs. Further research is needed to improve imputation from low-density chips as well as strategies to select optimal haplotype lengths and other options in findhap automatically.

Two stages of imputation, first from low to medium density and then from medium to high, can be more accurate than imputing directly from low densities to high. Two stages improved accuracy using findhap or FImpute to impute from low to medium and then to high-density array genotypes [14] or using Beagle to impute from medium to high density and then to sequence data [5]. However, the choice of which medium density to choose is rapidly becoming more difficult because genotypes from 18 different arrays of varying densities are used routinely. The current focus was mainly on HD to low-coverage sequence data.

Population size, sequence read depth and error rate all affected the accuracy of genotype calling and imputation. Druet et al. [9] also examined tradeoffs for a given total cost of more coverage versus more individuals using simulated bull sequences and Beagle. Loci with moderate frequency were discovered and imputed accurately using only 50 bulls sequenced at 12× coverage, but accuracy decreased dramatically for loci with low to very low frequencies. Use of only 25 bulls with 24× coverage reduced accuracy because many variants were not detected, and imputation was poor for loci with very low minor allele frequency when too few haplotypes were observed. When the sequencing effort was constant at 600-fold, accuracy improved using more individuals sequenced at lower coverage (e.g. 150 individuals with 4× coverage instead of 75 individuals with 8× coverage). With 1200-fold total coverage, sequencing 200 individuals with 6× coverage was better than 100 with 12×, and 400 with 3× was better than 200 with 6 ×.

Our results are consistent with Druet et al. [9] but also include lower coverage (<4×) and larger populations (≤10,000). For constant cost of sequencing, our results showed optimum read depth was only 1× with 500-fold total coverage, 2× with 1000-fold total coverage and 4× with 2000-fold total coverage for imputation from HD arrays (Fig. 3). For a given total sequencing effort, higher read depths were desired if the goal was direct investigation of the sequenced individuals, but larger populations with lower coverage of 2× to 4× were desired for imputation from HD arrays.

For a newly discovered quantitative trait locus (QTL) to explain more variance than the best markers already genotyped in the population, the QTL imputation accuracy must be fairly high and exceed the correlation between the QTL and the markers. For example, an observed marker with no imputation loss and correlated by >0.90 to the QTL may have larger estimated effect than the true QTL imputed with <0.90 accuracy. However, if the true QTLs can be located, they can be added to arrays to avoid imputation in the future. Our accuracies from simulated data are higher than those of Brøndum et al. [8] from real data, perhaps because they computed correlations at each locus and then averaged, which puts more emphasis on low-MAF markers that are more difficult to impute [35]. Monomorphic loci do not contribute to correlations after centering because such loci add zero to both numerator and denominator.

Sequence analysis begins by detecting sites that exhibit evidence of polymorphism and then calling or imputing genotypes at the polymorphic sites [25]. We did not directly examine variant detection, but findhap does estimate allele frequencies iteratively beginning with simple allele counts. As allele probabilities are updated within each haplotype, allele frequencies can approach zero when the observed number of minor alleles is less than or equals the expected number based on the error rate. Thus, SNV selection is somewhat automatic when imputing with findhap if all of the original polymorphic sites are included in the data. However, only two alleles per site are allowed, and other observed alleles may be treated as errors.

The sequence genotypes of human chromosome 22 from the 1000 Genomes Project [1] were converted to differing read depths and error rates. Genotype calling and imputation results were not as accurate with actual human as with the simulated bovine sequences, probably because the simulation was based on a real Holstein pedigree with much higher average relationships than in most human studies. Finding long, shared haplotypes is much easier when close relatives appear in the reference population. In many human datasets, the shared haplotypes are shorter and originate from shared ancestors many generations ago. This probably caused lower imputation accuracy from array to sequence data in humans than in cattle even though a denser array was simulated for humans. Because genotypes are imputed from haplotypes in both findhap and Beagle, improved imputation accuracy implies either that more alleles within the haplotype are correct or that the correct haplotypes are selected more often. Direct comparisons of the haplotypes could be helpful but were not examined here.

Computation is a big challenge as numbers of genotyped and sequenced animals both grow. For imputing genotypes from low-density (3000 to 10 K SNPs) to medium-density (50,000 SNPs) arrays using populations of >10,000 animals, version 2 of findhap was about as accurate as version 3 of Beagle but 50 times faster and with much less memory required [10, 36]. The current US dairy cattle database has >3000 animals genotyped with HD, >150,000 with 50,000 SNPs, >500,000 with lower densities (3000 to 16,000 SNPs) and >30 million phenotyped animals. Holstein reference populations in Europe and North America already include >25,000 bulls genotyped with ≥50,000 SNPs, and some also include many more cows. Imputation of 30 million SNVs on all 30 chromosomes for 25,000 reference bulls requires about 9 days with 25 processors as tested using Intel Nehalem-EX 2.27 GHz processors with version 4 of findhap but could take many months with Beagle. Therefore, Beagle’s use is restricted to smaller populations or fewer variants. Version 4 of findhap retains the efficient computing time and memory of previous versions and allows accurate imputation using low-coverage sequence data.

## Conclusions

The algorithm for simultaneous genotype calling from sequence data and imputation from array genotypes of various densities in findhap (version 4) is very efficient. With low read depths or high error rates, updating allele probabilities within pairs of haplotypes for each individual is more accurate than first calling genotypes or computing genotype probabilities and then imputing. Accuracy of genotype calling and imputation both improved if reference populations with low-coverage sequence data also were genotyped with HD arrays, but accuracy decreased slightly when lower-density array genotypes were combined with low-coverage sequence data because haplotype length options were not optimal for all data inputs. Use of pedigree can greatly speed imputation by first examining haplotypes of known ancestors and improves probability of finding the correct haplotypes. More efficient imputation allows geneticists to locate and test effects of more DNA variants from more individuals and to include those in future prediction and selection.

## Methods

Tests of genotype calling and imputation used up to 10,000 simulated bovine sequences containing 1 million SNVs on a chromosome of average length (100 cM) or 1092 actual human sequences containing 394,724 SNVs on the shortest chromosome (HSA 22). Simulated array genotypes contained 2 % of the simulated bovine SNVs or 10 % of the total human SNVs. Imputation accuracy was measured as the correlation of imputed with true genotypes or percentage of genotypes that were correctly called. The parameters tested were read depth, error rate, reference population size, and inclusion or exclusion of HD genotypes in the sequence data as summarized in Table 6.

**Table 6:** Parameters tested for genotype calling and imputation from an HD array

Dataset	Sequenced population					Array genotypes	
	SNVs	Population size	Sequence read depth	Error rate (%)	HD information included	SNPs/SNVs	Population size
Bovine (simulated)	1,000,000	250	1×, 2×, 4×, 8×, 16×	0, 1, 4, 16	No	20,000	250
					Yes	20,000	250
		500	1×, 2×, 4×, 8×, 16×	0, 1, 4, 16	Yes	20,000	250
		1000	1×, 2×, 4×, 8×, 16×	0, 1, 4, 16	Yes	20,000	250
		10,000	1×, 2×	1	Yes	20,000	250
		1000	8×	1	Yes	3333	250
		1000	8×	1	Yes	1× sequence	250
						2× sequence	250
Human (chromosome 22)	394,724	884	1×, 2×, 4×, 8×, 16×	0, 1, 4, 16	Yes	39,440	218

### Simulated bovine sequences

The bovine pedigree and the number of SNVs are identical to those in a previous test of sequence imputation [14], but the simulated SNVs have reduced read depth and more errors instead of directly observed genotypes. The pedigree contained 250 randomly chosen US Holstein bulls born in 2010 and about 10 generations of their known ancestors for a total of 23,656 animals. The top 250, 500, 1000 or 10,000 older bulls with the most US daughters were given sequence data and used to test the accuracy of genotype calling as well as serve as the reference population for imputation. The younger 250 bulls were used to test imputation from genotypes from different density arrays (600 K, 60 K and 10 K) and low-coverage sequence data. The ancestor bulls were highly selected, had an average birth year of 1987, and sometimes were separated from the younger bulls by several generations because the generation interval was about 5 years.

One simulated chromosome with 1 million polymorphic loci and a length of 100 cM represented the bovine genome, which has 30 chromosome pairs and about 30 million SNVs [3]. The simulated 600 K, 60 K and 10 K chips contained subsets of 20,000, 2000 and 333 markers, respectively. The markers placed on chips had allele frequencies (*p*) that were uniformly distributed between zero and one in the founding population, but the sequence variants had lower minor allele frequencies and a quadratic distribution generated from the uniform distribution by

$$ \frac{0.5\pm 0.25\sqrt{\left|\mathrm{uniform}-0.5\right|}}{\sqrt{0.5}} $$

where the term after the ± sign was added if the uniform was >0.5 or subtracted if ≤0.5. Haplotypes from each unknown parent of the known ancestors were generated with linkage disequilibrium and then inherited with recombination to descendants. All simulated markers and SNVs were biallelic. Maternal or paternal alleles had equal likelihood to be read.

The simulated read depths had Poisson distributions with averages of 16, 8, 4, 2 or 1, representing medium to low-coverage sequence data. Read depth for the sequenced animals was reduced either at all variants or at all variants except the chip loci, which were given a read depth of 32. This procedure tested if sequence reads and HD arrays should be combined to improve imputation as recommended by Menelaou and Marchini [24]. Lower accuracy could be assumed for the array genotypes by either reducing the read depth or increasing the read error assumed for those loci, but simulated array genotypes had an error rate of 0.001 in this study.

The individual sequence reads contained 0, 1, 4 or 16 % error to test sensitivity to data quality. Another test included low-coverage sequence for young animals as proposed by De Donato et al. [37] and Hickey [38], but the sequence data for those animals also included 600 K array data. Imputation accuracy was measured as the percentage of matching true and called genotypes across all loci and also as the correlation between true and called genotypes after centering both by subtracting twice the frequency of the reference allele from the genotypes originally coded as 0, 1, and 2. Many previous studies with array data tested the percentage of correctly imputed genotypes, but correlations or squared correlations are better for testing low frequency SNVs [34]. Calus et al. [35] also recommended scaling the genotypes to put more emphasis on low-MAF loci, but we did not divide by the genotype standard deviation before computing the correlations because the formula becomes undefined with monomorphic loci and because standardization is not used when we estimate marker effects.

### Human genome sequences

The human dataset contained actual sequence genotypes for 394,724 SNVs from the shortest human chromosome (22). The full human genome from The 1000 Genomes Project Consortium [1] contains approximately 30 million SNVs. Available data for 1092 human genomes were randomly divided into 884 for reference and 218 with simulated chip genotypes to test imputation. The HD genotypes contained 39,440 (10 %) of the total variants. The selected SNPs were those with highest minor allele frequency (>0.12) after first eliminating redundant SNPs (those with an absolute correlation of >0.90), removing SNPs closer than 100 bp and inserting other SNPs to close gaps of >500 bp. The result was roughly equivalent to a chip with approximately 3 million SNPs across all chromosomes, which was constructed with higher density than the 777,000-SNP Illumina BovineHD BeadChip to partially compensate for the much lower linkage disequilibrium in human populations.

### Haplotype probabilities

Imputation software can store allele probabilities within haplotypes to account for reduced read depth and high error rates, whereas previous software often stored only “known” alleles. New computer algorithms introduced in version 4 of findhap compute and store such probabilities to impute genotypes from sequence data and maintain many of the strategies and efficiencies from previous versions of findhap [31]. Phasing of known genotypes into haplotypes can be simple if parents and progeny are genotyped, but it is somewhat more difficult if the individuals are less related or their genotypes are known less precisely.

In findhap, individuals are processed from oldest to youngest, and known haplotypes of close ancestors are checked first to improve speed and accuracy. If ancestor haplotypes are not available or do not match the genotype, the most frequent haplotype that does not conflict with the genotype is selected from a list of all haplotypes sorted in descending frequency order. Alleles in the selected haplotype are removed from the genotype to obtain the complement haplotype. For example, if the genotype is AB and the selected haplotype is known to contain a B at that locus, the complement must contain an A. Then the next most frequent haplotype that agrees with the complement is located, and information from the genotype is used to update the two selected haplotypes. In previous versions of findhap, missing alleles were filled in the two haplotypes if the genotype was homozygous or if the genotype was heterozygous and the other haplotype allele was known. Version 4 uses probabilities instead of known alleles to select and compare haplotypes.

Probabilities within the two haplotypes of each individual are updated by applying conditional probability rules (Bayes theorem) to include the new information provided by that individual’s sequence data (S). The most likely haplotype (H1) and its complement (H2) are selected from a list, or a new haplotype is added if none in the list has a likelihood ratio higher than a minimum value determined from the number of loci. Prior probabilities for new haplotypes are set to allele frequency. Then posterior probabilities that the alleles h1 and h2 at a particular locus within H1 and H2, respectively, contain allele A are calculated from their prior probabilities (*p*_1_ and *p*_2_) and S at that locus. This updating process is repeated for each individual using the posterior probabilities in H1 and H2 as prior probabilities for the next individual with either of those same haplotypes. This procedure accumulates linkage information into the haplotype list instead of using multi-locus math to account for linkage as in Duitama et al. [27].

The sequence observations are coded simply as the numbers of A (*n*_A_) and B (*n*_B_) alleles observed; the three main categories of observed data are only *n*_A_, only *n*_B_ or both *n*_A_ and *n*_B_ positive. With no sequencing error, the third category always indicates a heterozygote; however, the first two categories do not always indicate homozygotes because heterozygotes also produce only *n*_A_ or only *n*_B_ observations at a rate of 0.5(*n*_A_ + *n*_B_) each. For example, *n*_A_ = 4 and *n*_B_ = 0 could result from an AA homozygote that produced A alleles every time or an AB heterozygote that produced only A alleles with frequency of 0.5^4^ = 0.0625. With low-coverage sequence, *n*_A_ and *n*_B_ may be zero at many loci, and those are treated as missing observations. Storage of *n*_A_ and *n*_B_ is more efficient than storing the three genotype probabilities used in other software. Either strategy assumes that one reference and one alternate nucleotide are valid; reads of the other two nucleotides are considered errors and are treated as unknown.

Prior probabilities that the two haplotypes contain an A at a particular locus are *P*(h1 = A) and *P*(h2 = A), and both initially are set to allele frequencies before processing the first individual. Posterior probabilities *P*(h1 = A|*n*_A_, *n*_B_) and *P*(h2 = A|*n*_A_, *n*_B_) are then obtained by jointly accounting for the two prior probabilities and using the standard conditional probability rule such as in Duitama et al. [27]:

$$ P\left(\mathrm{h}1=\mathrm{A}\Big|{n}_{\mathrm{A}},{n}_{\mathrm{B}}\right)=P\left({n}_{\mathrm{A}},{n}_{\mathrm{B}}\Big|\mathrm{h}1=\mathrm{A}\right)\frac{P\left(\mathrm{h}1=\mathrm{A}\right)}{P\left({n}_{\mathrm{A}},{n}_{\mathrm{B}}\right)}; $$

$$ P\left(\mathrm{h}2=\mathrm{A}\Big|{n}_{\mathrm{A}},{n}_{\mathrm{B}}\right)=P\left({n}_{\mathrm{A}},{n}_{\mathrm{B}}\Big|\mathrm{h}2=\mathrm{A}\right)\frac{P\left(\mathrm{h}2=\mathrm{A}\right)}{P\left({n}_{\mathrm{A}},{n}_{\mathrm{B}}\right)}. $$

Posterior probabilities account for the error in individual sequence reads (errate) using math similar to that of Druet et al. [9], except that the probabilities are applied directly to haplotypes instead of first calling genotypes. For efficiency, probabilities of observing *n*_A_ and *n*_B_ given the three genotypes are calculated and stored for later use when processing each potential haplotype. Factorial terms in the binomial distribution are not stored because they always cancel when computing likelihood ratios:

$$ P\left({n}_{\mathrm{A}},{n}_{\mathrm{B}}\Big|\mathrm{AA}\right)=\frac{{\mathrm{errrate}}^{n_{\mathrm{B}}}\left(1-{\mathrm{errate}}^{n_{\mathrm{A}}}\right)\left({n}_{\mathrm{A}}+{n}_{\mathrm{B}}\right)!}{n_{\mathrm{A}}!{n}_{\mathrm{B}}\kern0.1em !}; $$

$$ P\left({n}_{\mathrm{A}},{n}_{\mathrm{B}}\Big|\mathrm{B}\mathrm{B}\right)=\frac{{\mathrm{errrate}}^{n_{\mathrm{A}}}\left(1-{\mathrm{errate}}^{n_{\mathrm{B}}}\right)\left({n}_{\mathrm{A}}+{n}_{\mathrm{B}}\right)!}{n_{\mathrm{A}}!{n}_{\mathrm{B}}\kern0.1em !}; $$

$$ P\left({n}_{\mathrm{A}},{n}_{\mathrm{B}}\Big|\mathrm{AB}\right)=\frac{0.5^{\left({n}_{\mathrm{A}}+\kern0.5em {n}_{\mathrm{B}}\right)}\left({n}_{\mathrm{A}}+{n}_{\mathrm{B}}\right)!}{n_{\mathrm{A}}!{n}_{\mathrm{B}}\kern0.1em !}. $$

The two prior probabilities *P*(h1 = A) and *P*(h2 = A) are labeled *p*_1_ and *p*_2_, respectively, for simplicity. From these, the conditional probability of observing *n*_A_ and *n*_B_ given that h1 contains A or that h2 contains A are calculated from

$$ P\left({n}_{\mathrm{A}},{n}_{\mathrm{B}}\Big|\mathrm{h}1=\mathrm{A}\right)={p}_2P\left({n}_{\mathrm{A}},{n}_{\mathrm{B}}\Big|\mathrm{AA}\right)+\left(1-{p}_2\right)P\left({n}_{\mathrm{A}},{n}_{\mathrm{B}}\Big|\mathrm{AB}\right); $$

$$ P\left({n}_{\mathrm{A}},{n}_{\mathrm{B}}\Big|\mathrm{h}2=\mathrm{A}\right)={p}_1P\left({n}_{\mathrm{A}},{n}_{\mathrm{B}}\Big|\mathrm{AA}\right)+\left(1-{p}_1\right)P\left({n}_{\mathrm{A}},{n}_{\mathrm{B}}\Big|\mathrm{AB}\right). $$

The unconditional probability of observing *n*_A_ and *n*_B_ is computed by summing probabilities that the true genotype was AA, BB or AB multiplied by the probability of observing *n*_A_ and *n*_B_ given each genotype. The overall probability for the population uses the same formula except that the population frequency *p* is substituted for the haplotype prior probabilities *p*_1_and *p*_2_:

$$ \begin{array}{c}P\left({n}_{\mathrm{A}},{n}_{\mathrm{B}}\right)={p}_1{p}_2P\left({n}_{\mathrm{A}},{n}_{\mathrm{B}}\Big|\mathrm{AA}\right)+\left(1-{p}_1\right)\left(1-{p}_2\right)P\left({n}_{\mathrm{A}},{n}_{\mathrm{B}}\Big|\mathrm{B}\mathrm{B}\right)\\ {}+\kern0.5em \left({p}_1+{p}_2-2{p}_1{p}_2\right)P\left({n}_{\mathrm{A}},{n}_{\mathrm{B}}\Big|\mathrm{AB}\right).\end{array} $$

The H1 and H2 mostly likely to form the genotype are selected using likelihood ratio tests from a haplotype list that is sorted by descending frequency. The probability of observing s at each locus is divided by the probability that s would be observed if alleles were chosen randomly from the population (*p*_2_ = *p*), and these ratios at each locus in a potential H1 are multiplied to obtain the joint likelihood ratio $\frac{P\left(\mathrm{S}\Big|\mathrm{H}1\right)}{P\left(\mathrm{S}\right)}.$ A particular haplotype H1 is selected if the joint likelihood ratio is >1/*n*, where *n* is the number of loci with observed data in the haplotype. The H2 is selected based on the joint likelihood of S given H2 and H1 divided by the likelihood given H1; i.e.

$$ \frac{P\left(\mathrm{S}\Big|\mathrm{H}1,\mathrm{H}2\right)}{P\left(\mathrm{S}\Big|\mathrm{H}1\right)}>\frac{1}{n\;\left[1+\left(n\kern0.1em /\kern0.1em 100\right)\right]}. $$

If the likelihood ratio is <1/*n* at any individual locus, the haplotype is discarded immediately to save computation.

The two selected H1 and H2 are updated by combining the formulas above to obtain their posterior probabilities of containing allele A given the sequence data and the two haplotype prior probabilities at each locus:

$$ P\left(\mathrm{h}1=\mathrm{A}\;\Big|{n}_{\mathrm{A}},{n}_{\mathrm{B}}\right) = \frac{\left[{p}_2P\left({n}_{\mathrm{A}},{n}_{\mathrm{B}}\;\Big|\mathrm{AA}\right)+\left(1-{p}_2\right)P\left({n}_{\mathrm{A}},{n}_{\mathrm{B}}\;\Big|\mathrm{AB}\right)\right]{p}_1}{p_1{p}_2P\left({n}_{\mathrm{A}},{n}_{\mathrm{B}}\;\Big|\mathrm{AA}\right)+\left(1-{p}_1\right)\left(1-{p}_2\right)P\left({n}_{\mathrm{A}},{n}_{\mathrm{B}}\;\Big|\mathrm{B}\mathrm{B}\right) + \kern0.5em \left({p}_1+{p}_2-2{p}_1{p}_2\right)P\left({n}_{\mathrm{A}},{n}_{\mathrm{B}}\;\Big|\mathrm{AB}\right)}; $$

$$ P\left(\mathrm{h}2=\mathrm{A}\;\Big|{n}_{\mathrm{A}},{n}_{\mathrm{B}}\right) = \frac{\left[{p}_1P\left({n}_{\mathrm{A}},{n}_{\mathrm{B}}\;\Big|\mathrm{AA}\right)+\left(1-{p}_1\right)P\left({n}_{\mathrm{A}},{n}_{\mathrm{B}}\;\Big|\mathrm{AB}\right)\right]{p}_2}{p_1{p}_2P\left({n}_{\mathrm{A}},{n}_{\mathrm{B}}\;\Big|\mathrm{AA}\right)+\left(1-{p}_1\right)\left(1-{p}_2\right)P\left({n}_{\mathrm{A}},{n}_{\mathrm{B}}\;\Big|\mathrm{B}\mathrm{B}\right) + \kern1em \left({p}_1+{p}_2-2{p}_1{p}_2\right)P\left({n}_{\mathrm{A}},{n}_{\mathrm{B}}\;\Big|\mathrm{AB}\right)}. $$

### Genotype probabilities

To compare findhap with Beagle, genotype probabilities for sequenced animals were calculated because the input file for Beagle requires genotypes or genotype probabilities:

$$ P\left(\mathrm{AA}\Big|{n}_{\mathrm{A}},{n}_{\mathrm{B}}\right)=\frac{P\left({n}_{\mathrm{A}},{n}_{\mathrm{B}}\Big|\mathrm{AA}\right)P\left(\mathrm{AA}\right)}{P\left({n}_{\mathrm{A}},{n}_{\mathrm{B}}\right)}; $$

$$ P\left(\mathrm{AB}\Big|{n}_{\mathrm{A}},{n}_{\mathrm{B}}\right)=\frac{P\left({n}_{\mathrm{A}},{n}_{\mathrm{B}}\Big|\mathrm{AB}\right)P\left(\mathrm{AB}\right)}{P\left({n}_{\mathrm{A}},{n}_{\mathrm{B}}\right)}; $$

$$ P\left(\mathrm{B}\mathrm{B}\Big|{n}_{\mathrm{A}},{n}_{\mathrm{B}}\right)=\frac{P\left({n}_{\mathrm{A}},{n}_{\mathrm{B}}\Big|\mathrm{B}\mathrm{B}\right)P\left(\mathrm{B}\mathrm{B}\right)}{P\left({n}_{\mathrm{A}},{n}_{\mathrm{B}}\right)} $$

where the genotype probabilities *P*(AA), *P*(AB) and *P*(BB) were set to (1 − *p*)^2^, 2*p*(1 − *p*) and *p*^2^, respectively, with Hardy-Weinberg equilibrium assumed.

### Availability of supporting data

Example files of simulated bovine data and the programs used for simulating true genotypes, converting genotypes to sequence read depths, and imputing genotypes are freely available from the Animal Improvement Program web site (http://aipl.arsusda.gov/software/software.html). The genotypes from human chromosome 22 are available from the 1000 genomes sequence imputation panel at http://csg.sph.umich.edu/abecasis/MACH/download/1000G.2012-03-14.html.

## Abbreviations

HD: High density
MAF: Minor allele frequency
QTL: Quantitative trait locus
SNP: Single nucleotide polymorphism
SNV: Single nucleotide variant
600K: 600,000
60K: 60,000
10K: 10,000
